# Transcriptional regulation of heat shock proteins and ascorbate peroxidase by CtHsfA2b from African bermudagrass conferring heat tolerance in *Arabidopsis*

**DOI:** 10.1038/srep28021

**Published:** 2016-06-20

**Authors:** Xiuyun Wang, Wanlu Huang, Zhimin Yang, Jun Liu, Bingru Huang

**Affiliations:** 1 College of Agro-grassland Science, Nanjing Agricultural University, Nanjing, 210095, China; 2 Department of Plant Biology and Pathology, Rutgers, the State University of New Jersey, New Brunswick, NJ, 08901, USA

## Abstract

Heat stress transcription factor A2s (HsfA2s) are key regulators in plant response to high temperature. Our objectives were to isolate an *HsfA2* gene (*CtHsfA2b*) from a warm-season grass species, African bermudagrass (*Cynodon transvaalensis* Burtt-Davy), and to determine the physiological functions and transcriptional regulation of *HsfA2* for improving heat tolerance. Gene expression analysis revealed that *CtHsfA2b* was heat-inducible and exhibited rapid response to increasing temperature. Ectopic expression of *CtHsfA2b* improved heat tolerance in *Arabidopsis* and restored heat-sensitive defects of *Arabidopsis hsfa2* mutant, which was demonstrated by higher survival rate and photosynthetic parameters, and lower electrolyte leakage in transgenic plants compared to the WT or *hsfa2* mutant. *CtHsfA2b* transgenic plants showed elevated transcriptional regulation of several downstream genes, including those encoding ascorbate peroxidase (*AtApx2*) and heat shock proteins [*AtHsp18.1-CI*, *AtHsp22.0-ER*, *AtHsp25.3-P* and *AtHsp26.5-P(r)*, *AtHsp70b* and *AtHsp101-3*]. *CtHsfA2b* was found to bind to the heat shock element (HSE) on the promoter of *AtApx2* and enhanced transcriptional activity of *AtApx2*. These results suggested that *CtHsfA2b* could play positive roles in heat protection by up-regulating antioxidant defense and chaperoning mechanisms. *CtHsfA2b* has the potential to be used as a candidate gene to genetically modify cool-season species for improving heat tolerance.

Temperatures above the normal optimum, sensed as heat stress (HS), are main environmental adverse factors for terrestrial plants limiting their growth, yield and distribution worldwide. Abiotic stress, including HS, affects the stability of membranes, proteins and enzymatic reactions, which subsequently disrupts the metabolic balance that causes the accumulation of toxic products, such as reactive oxygen species (ROS)^1^. As sessile organisms, plants have specialized systems to maintain growth and metabolic activities with signal transduction pathways, heat stress transcription factors (HSFs), heat shock proteins (HSPs) and antioxidative substances assembled to form the complex networks of heat stress response (HSR) responsible for heat tolerance in plant species^1^,^2^.

As the terminal components of the heat stress signal transduction chain, HSFs bind to the heat shock elements (HSEs) present in the promoter of downstream heat-inducible genes and play a central role in the HSR^3^,^4^. Compared to Yeast (*Saccharomyces cerevisiae*), fruit fly (*Drosophila melanogaster*) and mammals, plants have a larger HSF family involved in the activation of complex HSR networks due to their sessile characteristic. There are 26 HSF members in rice (*Oryza sativa*)^5^ and 21 HSF members in *Arabidopsis thaliana*^6^. However, recent studies have demonstrated that among the multiple members of HSF family in higher plants, the A1- and A2-type HSFs play important roles in the HSR^7^,^8^. HsfA1a was considered as the master regulator, but the expression patterns indicate that the HsfA1 group was constitutively expressed at low levels in most organs^9^,^10^. HsfA2s are the most strongly-induced proteins during HS and are believed to be one of the key regulators of plant stress responses to HS^11^,^12^. In addition, it has been suggested that constitutively-expressed *AtHsfA1a* and *b* are required for the early transcription of HS-associated genes, whereas heat-inducible *AtHsfA2* is the major factor of the subsequent HSR in *Arabidopsis*^13^,^14^. Moreover, thermotolerance of tomato (*Lycopersicon esculentum*) protoplast with silenced *LeHsfA1* was restored by *LeHsfA2*, which resulted in expression of heat-related proteins and assembly of heat stress granules^9^.

Recent studies demonstrated that the functions of the same type of HSFs have a degree of redundancy in regulating HSR. There was no influence on heat tolerance in single knock-out (KO) mutant of *AtHsfA1s*, little effects in double KO mutant and more effects in triple KO mutant, but complete loss of thermotolerance in quadruple KO mutant^15^. The same redundant conditions were also found in the four A2-type HSFs (OsHsfA2b, c, d and e) of rice that play similar roles in the response to HS^16^. However, overexpression of a member of the same type of HSFs, *HsfA1d* of *Thellungiella salsuginea*^17^ or *HsfA2e* of rice^16^ improved the thermotolerance in transgenic plants. Furthermore, it has been demonstrated that improved stress tolerance is mainly associated with overexpression of HSF genes instead of knockout mutagenesis^16^.

Compared to model plants, there have been few studies investigating HSF-regulation of heat tolerance in perennial grass species which exhibit diverse levels of heat tolerance. Based on the optimal temperatures for growth and development, perennial grass species can be divided into two categories: warm-season and cool-season species with optimal temperatures in the range of 26–35 °C and 15–24 °C, respectively, and therefore, warm-season grass species have superior heat tolerance, compared to cool-season species^18^. We hypothesized that *HsfA2* gene from warm-season grass plants could play positive roles in heat tolerance by regulating defense mechanisms, such as the induction of genes for antioxidant and heat shock protection. The objectives of the study were to isolate an *HsfA2* gene (*CtHsfA2b*) from a heat-tolerant warm-season perennial grass species, African bermudagrass (*Cynodon transvaalensis* Burtt-Davy), and to determine the physiological functions and transcriptional regulation of *CtHsfA2b* on downstream target genes conferring heat tolerance in the model species, *Arabidopsis*. To our knowledge, this is the first study reporting the roles of *HsfA2b* cloned from a warm-season perennial grass species in improving plant heat tolerance through transcriptional upregulation of a chaperoning and antioxidant-defense gene in improving plant heat tolerance. *CtHsfA2b* could be used as a candidate gene to genetically modify cool-season grass species or developing molecular markers for improving cool-season grass heat tolerance in the future.

## Materials and Methods

### Growth conditions and stress treatment

Plants of African bermudagrass were collected from field plots at Nanjing Agricultural University and were propagated from stolons with at least one node per segment and placed into a hydroponic system with half-strength Hoagland’s nutrient solution^19^ on September 2, 2013. The nutrient solution was changed weekly to maintain adequate nutrient supply and aerated using air pumps to provide sufficient oxygen to the plants. Clonal plants were maintained in a growth chamber at 30/25 °C (day/night), 14 h photoperiod, 65% humidity and 650 μmol m^−2 ^s^−1^ photosynthetically active radiation for two weeks to allow for establishment.

To induce expression of heat-responsive genes, plants were subjected to heat stress at 42 °C for 6 h followed by recovery at 30 °C on September 15, 2013. Leaves and roots of African bermudagrass were collected at 0, 0.5, 1, 3, 6, 8, 12 and 24 h and flash frozen in liquid nitrogen for RNA extraction.

### Isolation of CtHsfA2b coding sequence and gene expression analysis

Due to the rapid response of *HsfA2* genes to heat stress^16^, African bermudagrass leaves exposed to 42 °C for 1 h were used for gene isolation. Total RNA was extracted using Tripure Isolation Reagent Kit (Roche Diagnostic, Basel, Switzerland) and cDNA was synthesized by a Transcriptor First Strand cDNA Synthesis Kit (Roche Diagnostic, Basel, Switzerland). One pair of primers (*CtHsfA2b*-F/R, see Supplementary Table S1) was used to isolate the 3′ region of *CtHsfA2b* cDNA sequence according to the expressed sequence tag (EST) database of bermudagrass^20^. The remaining sequence was obtained by using a SMARTer^®^ RACE 5′/3′Kit (Clontech Laboratories, Mountain View, CA, USA) with specific primers *CtHsfA2b*-GSP and -NP. The open reading frame (ORF) of *CtHsfA2b* was amplified using *CtHsfA2b*-S and *CtHsfA2b*-A primers. All positive clones confirmed by colony PCR were sequenced by Sangon Co. Ltd (Shanghai, China). Primers were designed by using Premier Primer 5.

For qRT-PCR analysis, total RNA was extracted using RNApure fast isolation Kit (YuanPingHao, Tianjin, China) and first strand cDNA was synthesized by the PrimeScript RT reagent Kit with gDNA Eraser (Perfect Real Time) (Takara, Otsu, Japan). qRT-PCR was performed with SYBR Green I Master reaction system (Roche Diagnostic, Rotkreuz, Switzerland) on Roche LightCycler480 II (Roche Diagnostic, Rotkreuz, Switzerland). Each sample was performed with three biological replicates, each with three technical replicates. Relative expression level of genes was determined by 2^−ΔΔCT^ method using *CtEF1α*^21^ and *AtActin2* as respective reference genes of African bermudagrass and *Arabidopsis*. Primers for qRT-PCR are listed in Supplementary Table S1.

### Plant expression vector construction and transformation

The ORF of *CtHsfA2b* with *Sal* I and *Not* I sites was first linked to a pENTR1A Dual Selection Vector and then transformed into a pEarleyGate 103^22^ destination plasmid using LR Clonase II enzyme mix (Invitrogen, Carlsbad, CA, USA). The destination plasmid was transformed into 8 *Arabidopsis* plants (T0) mediated by *Agrobacterium tumefaciens* using the floral dip method^23^.

For screening the first generation of transgenic lines (T1), more than 600 seeds collected from transformed *Arabidopsis* were sown on MS medium with 20 μg ml^−1^ glufosinate ammonium in October, 2013. Seventeen positive transgenic lines out of 20 surviving T1 seedlings were confirmed by PCR analysis using primers *CtHsfA2b*-S and pEarleyGate103-R. Hundreds of T2 seeds collected in January 2014 from T1 plants were also screened for green fluorescent protein (GFP) observation to remove the non-transgenic plants that derived from allele segregation. T2 resistant-seedlings that expressed the *CtHsfA2b* gene were used for further analysis.

### Confirmation of *CtHsfA2b* functions in improving heat tolerance in *Arabidopsis*

*Arabidopsis* ecotype Colombia was used as the wild type (WT). The *HsfA2* (At2g26150) T-DNA insertion mutant SALK_008978 (*hsfa2*)^24^ was obtained from the *Arabidopsis* Biological Resource Center (ABRC; Ohio State University). The T-DNA insertion and homozygous line were confirmed by PCR amplification with primer LP, RP and LBp1.3 (see Supplementary Table S1) and designed according to the SIGnAL website (http://signal.salk.edu/tdnaprimers.2.html).

To compare heat tolerance among WT, *hsfa2* and transgenic lines, *Arabidopsis* seeds were grown on Murashige and Skoog (MS) Medium under a 12-h photoperiod at 25 °C for 6 d. One set of the seedlings was used for protein localization using GFP observation and the examination of survival rates under heat treatment. Another set of plants was transferred to plastic pots (7 cm diameter and 7 cm depth) filled with a mixture of 25% vermiculite and 75% peat moss and maintained in a growth chamber at 25/20 °C (day/night), 14 h photoperiod, 65% humidity and 150 μmol m-2 s-1 photosynthetically active radiation for further examination of heat responses in mature plants and for gene expression analysis.

### Physiological measurements of *Arabidopsis*

30-day-old wild type and *35S:CtHsfA2b* transgenic *Arabidopsis* were treated with heat stress of 45/40 °C (day/night) for 72 h, and physiological measurements were performed after 7 d of recovery. Leaf net photosynthetic rate (Pn) was measured using a *Licor 6400* photosynthetic system (*LI-6400, Li-Cor*, Lincoln, NE, USA). Leaf photochemical efficiency was expressed as the variable fluorescence (Fv) to the maximal fluorescence (Fm) ratio (Fv/Fm) with a fluorescence induction monitor (OPTI-Sciences, Hudson, NY, USA) on dark-adapted leaves. Chlorophyll content was measured using a spectrophotometer (GE Healthcare Life Sciences, Cambridge, UK) after leaves were soaked in dimethylsulfoxide for 48 h according to previous methods^25^. Cellular membrane stability was estimated based on leaf electrolyte leakage (EL)^26^. The initial (Ci) and maximum (Cmax) levels of EL were measured using a conductance meter (Thermo Scientific, Beverly, MA, USA). Relative EL was calculated as EL = (Ci/Cmax) × 100.

### Subcellular localization of *CtHsfA2b*

The pENTR1A Dual Selection Vector containing *CtHsfA2b* was LR-recombined with a small binary vector p2GWF7.0 for fusion-expression of CtHsfA2b and enhanced green fluorescent protein (eGFP) in *Arabidopsis* protoplasts. Healthy and mature leaves of 4-week-old *Arabidopsis* were used for protoplast isolation. *Arabidopsis* protoplast isolation and transfection were performed according to a modified method^27^. The transfected protoplasts were mixed with DAPI solution (Yeasen Biotech Co., Ltd, Shanghai, China) to a final concentration of 10 μg/ml for a five-minute incubation at room temperature and then observed under a Confocal Laser Scanning Microscope (Carl Zeiss, Jena, Germany). In order to verify the permanent subcellular localization of CtHsfA2b, root tips of 3-day-old transgenic *Arabidopsis* seedlings grown on agar medium were cut and placed on slides for GFP observation using a fluorescence microscope BX53 (Olympus, Tokyo, Japan).

### Yeast one-hybrid (Y1H) analysis of CtHsfA2b binding to HSE

In order to determine DNA binding-ability of CtHsfA2b to HSEs on the promoter of *AtApx2* (*ProAtApx2*), an Y1H assay was performed according to manufacturer’s instructions using a One-Hybrid Library Construction & Screening Kit (Clontech Laboratories, Mountain View, CA, USA) with modification of pGADT7-Rec2 replaced by modified pGADT7 vector that had LR-recombination construction. For bait construction, three tandem HSE (TTTCtgGAAG) elements of the *AtApx2* promoter were linked to a pHis2.1 vector with *EcoR* I and *Sac* I sites and confirmed by sequencing with primers of pHis2.1-F/R. For the construction of the prey, the coding sequence of CtHsfA2b was LR-recombined into the pGADT7 vector. The bait pHis2.1-HSE and prey pGADT7-CtHsfA2b then were co-transformed into yeast strain Y187. Interaction between bait and prey was determined by cell growth on SD medium lacking Trp, Leu, and His (SD-WLH). Positive yeast clones verified by PCR were diluted by distilled water and potted on SD-WLH medium containing 50 mM 3-amino-1,2,4-triazole (3-AT, a competitive inhibitor of the HIS3 protein).

### Luciferase (LUC) assay of the binding of CtHsfA2b and *ProAtApx2 in vivo*

To further confirm the binding of CtHsfA2b and *ProAtApx2 in vivo*, the coding sequence of CtHsfA2b was transferred from a pENTR1A vector to a pMDC32 vector via a LR Clonase reaction. *ProAtApx2*, obtained with primers *ProAtApx2*-LUC-F/R, was introduced into a pCAMBIA 1381Z-LUC^28^ vector with *BamH* I and *Pst* I sites. Then, pMDC32-CtHsfA2b and pCAMBIA 1381Z-LUC-ProAtApx2 were transformed into *Arabidopsis* protoplasts with same transfection method described above. Beetle Luciferin Potassium Salt (Promega, Madison, Wisconsin, USA) was dissolved in sterile water to a final concentration of 7.8 mM and stored at −80 °C in separate aliquots. The stock solution was diluted 10 times before use. 10 μl of diluted beetle luciferin was mixed with 100 μl transfected protoplasts in each well of a 12-well strip. After incubation at 37 °C in the dark for 15 min, the luminescence was captured in a CCD Video Camera Imaging System (Vilber Lourmat, Marne la Vallée, France) and quantified by an ELIASA, Infinite 200 PRO (Tecan, Mannedorf, Switzerland).

### Detection of reactive oxygen species

Detection of superoxide free radicals was performed as described previously^29^ with minor modifications. Fifteen-day-old seedlings exposed to 25 °C or 37 °C for 1 h were harvested and then vacuum-infiltrated with 0.1 mg ml^−1^ nitroblue tetrazolium (NBT; Sigma,USA) in 25 mM HEPES buffer (pH 7.6). Samples were subsequently incubated at room temperature for 2 h in darkness. Then, chlorophyll was removed with 95% alcohol and photographs were taken with using a stereomicroscope (Olympus, Tokyo, Japan).

The accumulation of Hydrogen peroxide (H_2_O_2_) in *Arabidopsis* leaves was detected by 3,3′–diaminobenzidine (DAB; Sigma, USA) staining as described previously^29^ with minor modifications. Samples of the same batch of plants used for superoxide detection were vacuum-infiltrated with 0.1 mg ml^−1^ DAB in 50 mM Tris-acetate buffer (pH 5.0). Samples in DAB solutions were incubated at room temperature in the darkness for 24 h. Thereafter, chlorophyll was removed, and photos were taken as described above.

### Statistical analysis

The analysis of variance was performed to determine heat stress effects and variations among WT, transgenic plants and mutants in physiological parameters. The means were compared by Duncan and the Fisher’s protected least significance difference (LSD) test at a significance level of 0.05 by PASW statistics 18 (SPSS Inc, Chicago, IL, USA).

## Results

### Heat-inducible *CtHsfA2b* gene in African bermudagrass

**Figure 1:**
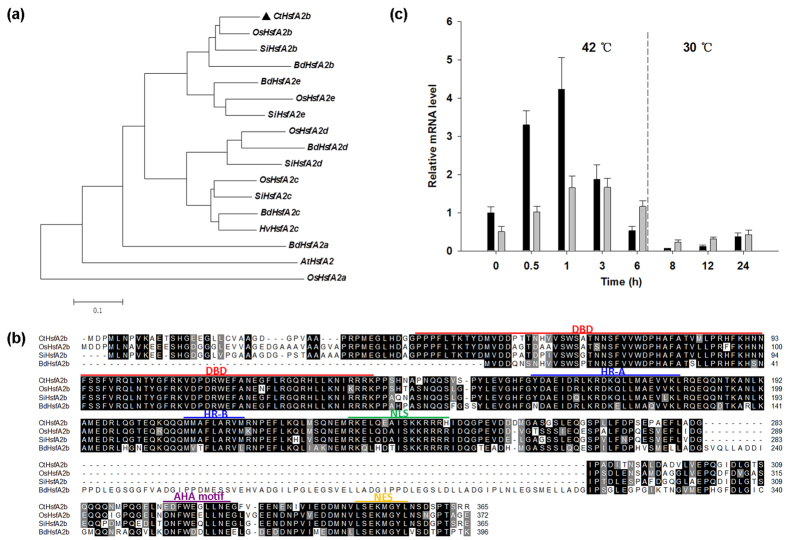
Characteristic analysis of CtHsfA2b. **(a)** Phylogeny analysis of the deduced amino acid sequences of CtHsfA2b and its homologous A2-type HSFs by the analysis of 1000 trees (bootstrap replicates) using neighbor-joining method of MEGA 4. Accession numbers of the proteins are as follows: CtHsfA2b (KU156630), OsHsfA2b (NP_001059028), SiHsfA2b (XP_004955620), BdHsfA2b (XP_010228292), BdHsfA2e (XP_003557472), OsHsfA2e (NP_001051552), SiHsfA2e (XP_004981438), OsHsfA2d (Q8H7Y6), BdHsfA2d (XP_003562044), SiHsfA2d (XP_004985605), OsHsfA2c (CAM32757), SiHsfA2c (XP_ 004983078), BdHsfA2c (XP_010234738), HvHsfA2c (BAJ89673), BdHsfA2a (XP_003559435), AtHsfA2 (NM_001124916), OsHsfA2a (NP_001044160). **(b)** Multiple sequence alignment and conserved domains of the four A2b-type HSFs in (**a**). Sequence alignment was performed with ClustalW Multiple alignment in BioEdit. Black regions indicate identical amino acids among the four sequences; gray regions indicate similar amino acids. DBD, DNA binding domain; HR-A and B, hydrophobic regions of oligomerization domain; NLS, nuclear localization signal; AHA motif, activator motif; NES, nuclear export signal. **(c)** Expression of CtHsfA2b in African bermudagrass leaves (black bars) and roots (gray bars) under heat stress (42 °C for 6 h) and recovery (30 °C) conditions, as determined by qRT-PCR. Data are presented as the mean values ± standard deviation (SD) of three biological replicates.

According to the bermudagrass EST database, we obtained a full-length sequence of *HsfA2* gene that consisted of 1386 bp nucleotides with a 1095 bp ORF encoding a 365-residue polypeptide via the RACE PCR method from African bermudagrass. The gene was classified into A2b subgroup of HSF by cluster analysis (Fig. 1a) and named African bermudagrass *HsfA2b* (*CtHsfA2b*, GenBank accession number: KU156630). Homologous alignment of the deduced amino acid sequences (Fig. 1b) revealed that *CtHsfA2b* had high sequence identity to *HsfA2b* of *Setaria italica*, *Oryza sativa* and *Brachypodium distachyon*, and it had a DNA binding domain (DBD), an oligomerization domain (OD) with a hydrophobic region (HR-A and B), a nuclear localization signal (NLS), a nuclear export signal (NES) and an activator motif (AHA motif).

qRT-PCR analysis demonstrated that *CtHsfA2b* exhibited rapid response to heat stress (Fig. 1c), as the expression level increased within 30 min and reached the highest level by 1 h of 42 °C heat treatment in leaves and roots of African bermudagrass. After reaching the highest expression level at 1 h, the gene expression attenuated in both leaves and roots and returned to the initial levels following recovery at 30 °C normal growth temperature.

### GFP-fused CtHsfA2b protein localized in nucleus

**Figure 2:**
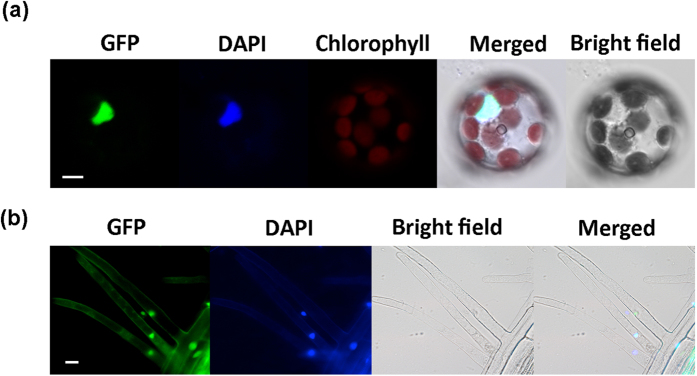
Subcellular localization of CtHsfA2b. **(a)** p2GWF7.0-CtHsfA2b-eGFP fusion protein localized in nucleus of isolated *Arabidopsis* protoplast. Bar = 5 μm. **(b)** pEarleyGate103-CtHsfA2b-GFP fusion protein localized in nucleus of root hair of *35S:CtHsfA2b* transgenic *Arabidopsis*. Bar = 20 μm. GFP, Green fluorescent protein; DAPI, 4′,6-diamidino-2-phenylindole.

In order to determine the cellular localization of CtHsfA2b, we first constructed a p2GWF7.0-CtHsfA2b-eGFP plasmid and transformed the plasmid into *Arabidopsis* mesophyll protoplasts. The completely-overlapped fluorescence signals of DAPI and GFP demonstrated that CtHsfA2b was localized to the nucleus (Fig. 2a). After obtaining CtHsfA2b transgenic *Arabidopsis* with the plant expression vector pEarleyGate 103 that also has a GFP encoding region, we further confirmed that CtHsfA2b protein was localized in the nucleus by scanning the roots of transgenic plants (Fig. 2b).

### Improved *Arabidopsis* heat tolerance through ectopic expression of CtHsfA2b

**Figure 3:**
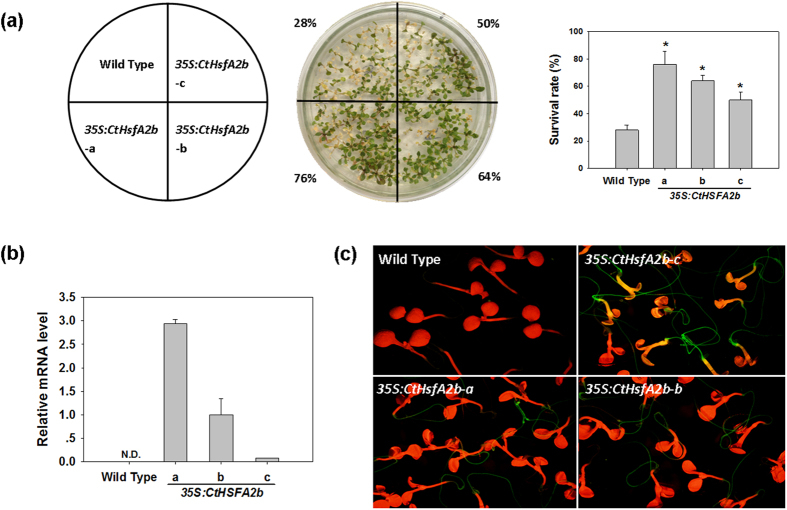
Ectopic expression of *CtHsfA2b* in *Arabidopsis*. **(a)** Heat stress tolerance of young seedlings of wild type and *35S:CtHsfA2b* transgenic lines. Each line of 30 seeds was grown on agar medium at 25 °C for 7 d and then placed into growth chamber maintained at 45 °C for 4 h. The photograph was taken 7 d after heat treatment. Values and the column chart indicate the total survival rate of plants in three petri dishes (replicates) for each line. Bars represent mean ± SD. Asterisk (*) indicates significant difference between each transgenic line and wild type according to Duncan and Fisher’s protected LSD test at a significance level of 0.05. **(b)** Relative *CtHsfA2b* gene expression in wild type and *35S:CtHsfA2b* transgenic plants by qRT-PCR. N.D., not detected. **(c)** Expression of CtHsfA2b-GFP fusion protein in *35S:CtHsfA2b* transgenic lines. Five-day-old seedlings on agar plate were observed under stereo fluorescence microscope.

To characterize the function of CtHsfA2b involved in heat tolerance, we transformed this gene into *Arabidopsis* (termed as *35S:CtHsfA2b*) for ectopic expression analysis. 7-day-old seedlings of transgenic lines (*35S:CtHsfA2b*-a, b and c) had higher survival rate (76%, 64% and 50% respectively) than the WT (28%) after 7 d recovery from 4 h heat stress at 45 °C (Fig. 3a). qRT-PCR analysis confirmed that *CtHsfA2b* was expressed only in the transgenic lines, but not in the WT (Fig. 3b). GFP-fused CtHsfA2b protein was also expressed in *35S:CtHsfA2b* transgenic lines (Fig. 3c). GFP exhibit bright green fluorescence in roots, but was not as obvious in leaves due to the interference by chlorophyll fluorescence.

**Figure 4:**
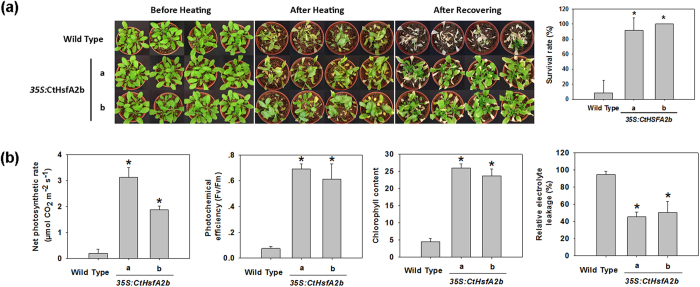
Heat tolerance and physiological measurements of *35S:CtHsfA2b* transgenic seedlings. **(a)** Phenotype of 30-day-old wild type and *35S:CtHsfA2b* transgenic plants before treatment, after heat stress at 45/40 °C (day/night) for 72 h and after 7 d recovery. The survival rates after recovery were presented on the right side. Asterisk (*) indicates significant difference between each transgenic line and wild type according to Duncan and Fisher’s protected LSD test at a significance level of 0.05. **(b)** Net photosynthetic rate, photochemical efficiency, chlorophyll content and relative electrolytic leakage of plants in (**a**) were measured after recovery. Data are expressed as the mean values ± SD of four biological replicates. Asterisk (*) indicates significant difference between each transgenic line and wild type plants according to Duncan and Fisher’s protected LSD test at a significance level of 0.05.

Ectopic expression of *CtHsfA2b* also improved tolerance to heat stress and recuperative ability of adult *Arabidopsis* after relief of heat stress. Leaves of both *35S:CtHsfA2b* transgenic lines and WT wilted after 3 d of 45/40 °C (day/night) heat stress, but the transgenic lines generated new leaves after returning to normal growth temperature for a week, whereas, the WT almost completely withered up and died with a significantly lower survival rate (Fig. 4a and see Supplementary Fig. S1). Net photosynthetic rate, photochemical efficiency and chlorophyll content of *35S:CtHsfA2b* transgenic lines were significantly higher than that of WT after heat treatment, which demonstrated more-stable photosynthetic system in the transgenic plants (Fig. 4b). In respect to cell membrane stability, *35S:CtHsfA2b* had less relative electrolyte leakage compared to the WT after heat stress.

### Potential target genes induced by CtHsfA2b in *Arabidopsis*

**Figure 5:**
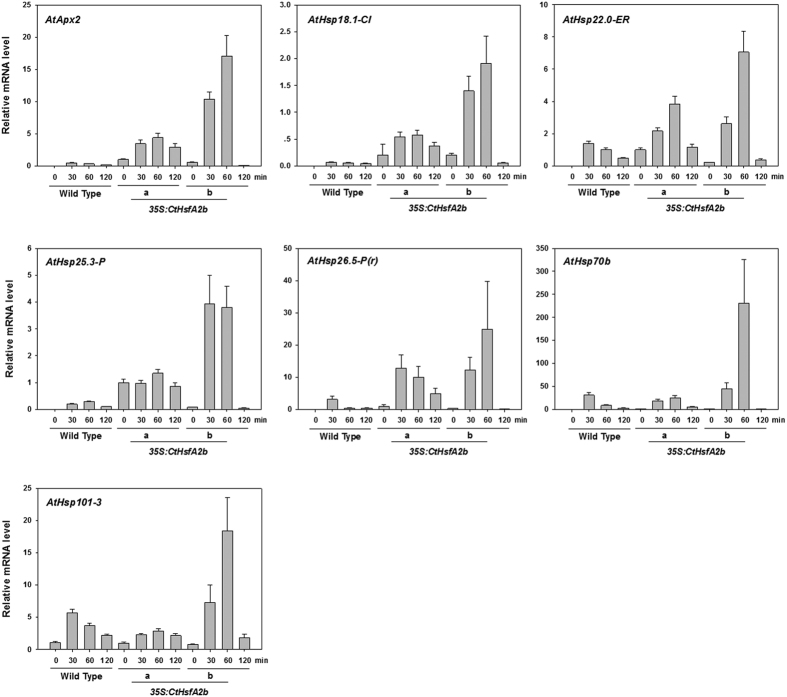
Expression analysis of potential target genes of CtHsfA2b. Leaves of wild type and *35S:CtHsfA2b* transgenic lines were sampled at 0, 30, 60 and 120 min during 2 h heat treatment at 37 °C for qRT-PCR. Data are expressed as the relative mean values ± SD of three biological replicates. Gene accession numbers and primers used for qRT-PCR are listed in Supplementary Table S1.

To investigate the underlying mechanisms by which ectopic expression of *CtHsfA2b* in Arabidopsis improved heat tolerance, the relative expression level of related genes was analyzed by qRT-PCR in transgenic lines and the WT plants under heat treatment. The results revealed that *AtApx2*, *AtHsp18.1-CI*, *AtHsp22.0-ER*, *AtHsp25.3-P*, *AtHsp26.5-P(r)*, *AtHsp70b* and *AtHsp101-3* were expressed in transgenic lines but not expressed or expressed at lower levels in the WT before heat stress (Fig. 5). After treatment at 37 °C, these genes were all up-regulated both in the WT and transgenic plants, but there was a greater extent of up-regulation of *AtApx2*, *AtHsp18.1-CI*, *AtHsp22.0-ER*, *AtHsp25.3-P* and *AtHsp26.5-P(r)* in transgenic lines compared to the WT. *35S:CtHsfA2b*-a, one of the transgenic lines, had equal or lower expression levels of *AtHsp70b* and *AtHsp101-3* than the WT, while *35S:CtHsfA2b*-b had greater up-regulation of these two genes. All the genes exhibited typical heat response characteristics in that the genes were induced by high temperature rapidly increased to the highest levels and then decreased to reduced levels within 2 h of heat treatment.

### Ectopic expression of CtHsfA2b rescued heat-sensitive defects of *Arabidopsis hsfa2* mutant

**Figure 6:**
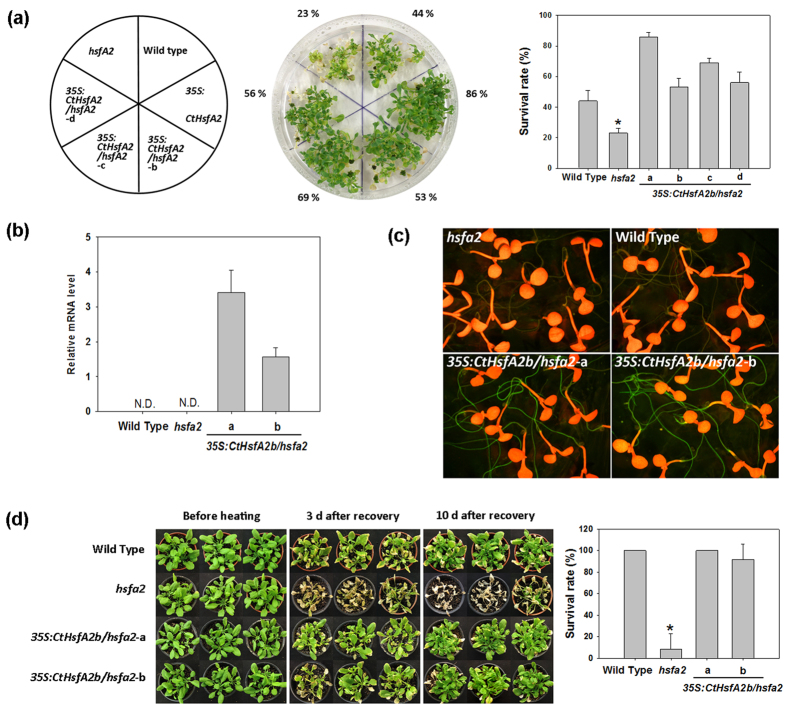
Complementary assay of *hsfa2* mutant transformed with *CtHsfA2b*. **(a)** Heat stress tolerance of seedlings of wild type, *hsfa2* and *35S:CtHsfA2b/hsfa2* transgenic lines. The experiments were performed as described in Fig. 3a, except that 16 seeds for each line were grown on one plate and 4 plates as replicates were treated at 45 °C for 3 h. Values and column chart indicate the total survival rate of plants in 4 plates for each line. Asterisk (*) indicates significant difference between the *hsfa2* mutant and others according to Duncan and Fisher’s protected LSD test at a significance level of 0.05. **(b)** Relative *CtHsfA2b* gene expression in wild type, *hsfa2* and *35S:CtHsfA2b/hsfa2* transgenic plants by qRT-PCR. N.D., not detected. **(c)** Expression of CtHsfA2b-GFP fusion protein in *35S:CtHsfA2b/hsfa2* transgenic lines. Details are the same as Fig. 3c. (**d**) Heat tolerance of *35S:CtHsfA2b/hsfa2* transgenic seedlings. The 30-day-old plants were treated at 45/40 °C (day/night) for 60 h, and recovered at 25 °C for 10 d. Survival rates after 10 d recovery were calculated. Bars represent mean ± SD. Asterisk (*) indicates significant difference between the *hsfa2* mutant and others according to Duncan and Fisher’s protected LSD test at a significance level of 0.05.

The positive roles of *CtHsfA2b* in improving plant tolerance to heat stress were further confirmed by ectopically expressing *CtHsfA2b* in the *Arabidopsis hsfa2* mutant (termed as *35S:CtHsfA2b/hsfa2*). The *hsfa2* mutant plants were severely injured with a lower survival rate (23%) than the WT (44%) after heat stress (Fig. 6a). However the *35S:CtHsfA2b/hsfa2* transgenic lines rescued *hsfa2* heat-sensitive defects and had a significantly higher survival rate (53–86%) than the WT. Results of qRT-PCR and GFP observation showed that the *CtHsfA2b* gene and its GFP-fused protein were expressed in *35S:CtHsfA2b/hsfa2*, but not in the *hsfa2* mutant and WT (Fig. 6b,c).

Adult seedlings grown in the soil suffered heat stress and were also examined, which confirmed that the heat treatment at 45/40 °C (day/night) for 60 h was lethal to the *hsfa2* mutant plants, but had less damage to WT and *35S:CtHsfA2b/hsfa2*, since *hsfa2* mutant plants did not recover even after relief of heat stress (Fig. 6d and see Supplementary Fig. S1). The survival rate of *hsfa2* mutant after 10 d recovery was significantly lower than WT and the transgenic lines. Ectopic expression of CtHsfA2b rescued the heat-sensitive phenotype of *hsfa2* mutant to the WT level at the mature seedling stage.

**Figure 7:**
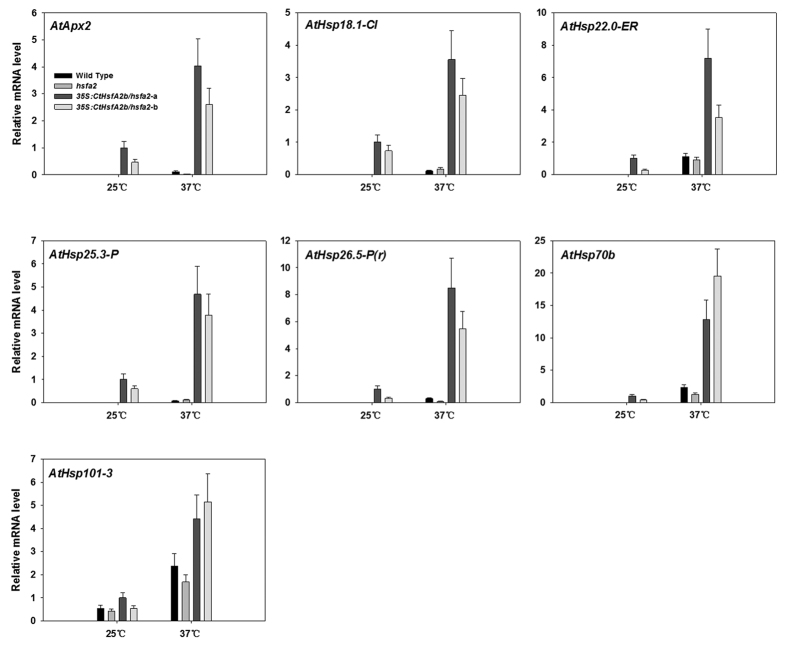
Expression analysis of potential target genes of CtHsfA2b in wild type, *hsfa2* and *35S:CtHsfA2b/hsfa2* transgenic lines. Plants were treated at 25 °C or 37 °C for 1 h and leaves were sampled for qRT-PCR. Data are expressed as the relative mean values ± SD of three biological replicates.

The expression of downstream genes was examined at 1 h of heat stress (37 °C). Under the control temperature condition (25 °C), these genes were all expressed in *35S:CtHsfA2b/hsfa2*, but most were not in the WT and *hsfa2* mutant plants, except *AtHsp101-3* (Fig. 7). All these genes were up-regulated in the WT, *hsfa2* and *35S:CtHsfA2b/hsfa2* after 1 h of heat treatment, but had higher transcript levels in the *35S:CtHsfA2b/hsfa2* transgenic lines compared to the WT and *hsfa2* mutant.

### CtHsfA2b binds the HSE on the promoter of *AtApx2*

**Figure 8:**
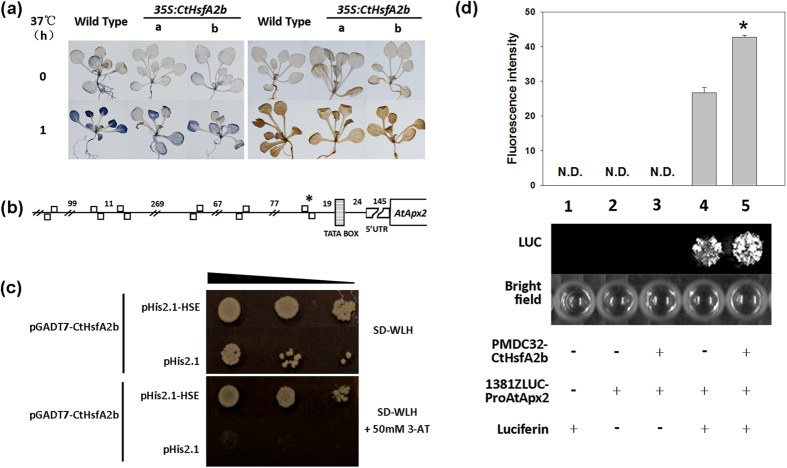
Detection of ROS and CtHsfA2b binding to *ProAtApx2*. **(a)** ROS detection of 15-day-old wild type and *35S:CtHsfA2b* transgenic plants after heat stress at 37 °C for 1 h. Nitroblue tetrazolium staining for superoxide free radicals (left panels) and 3,3′- diaminobenzidine staining for H_2_O_2_ (right panels). **(b)** Promoter structure of *AtApx2* with position of active HSE motifs. HSEs were drawn according to the previous nomenclature^6^: The open box above the line represents an active HSE head module and that below the line represents an active HSE tail module. *indicates the HSE used in the next Y1H binding assay. **(c)** Analysis of *CtHsfA2b* binding to the HSE on *ProAtApx2 in vitro* with Y1H system. The yeast strain Y187 was co-transformed with the bait (pHis2.1-HSE) and prey (pGADT7-CtHsfA2b) plasmids. The empty pHis2.1 vector was used as a negative control. **(d)** Analysis of *CtHsfA2b* binding to *ProAtApx2 in vivo* with using luciferase (LUC) assay in *Arabidopsis* protoplast. Signs of +/− indicates with/without the components listed on left side. *indicates significant difference of fluorescence intensity between lane 4 and 5 according to Student’s T-test at a significance level of 0.05.

We detected the ROS-scavenging ability that includes APX contribution under heat stress. The accumulation of superoxide radical and hydrogen peroxide in WT plants was higher than that in transgenic plants after 1 h of heat stress (Fig. 8a). Transcription levels of *AtApx2* were also up-regulated by ectopic expression of CtHsfA2b in *Arabidopsis*, as previously describe in Figs 5 and 7. Therefore, we examined CtHsfA2b potential binding to *ProAtApx2 in vitro* by using an Y1H assay with the typical active HSE motif on *ProAtApx2* (Fig. 8b) as bait. The results showed that CtHsfA2b could bind to the HSE on *ProAtApx2*, and then triggered the activation of the *HIS* reporter-gene expression on SD medium with WLH deficiency and even with 50 mM 3-AT added (Fig. 8c).

To further confirm the function of CtHsfA2b in regulation of *ProAtApx2 in vivo*, we performed a luciferase (LUC) assay in *Arabidopsis* protoplasts using the whole length sequence of *ProAtApx2* as the promoter of *LUC* reporter gene. The different negative controls (lane 1, 2 and 3) had no fluorescence (Fig. 8d). When 1381ZLUC-ProAtApx2 was transformed into protoplast with luciferin added as a substrate, obvious fluorescence was captured (Lane 4) because *ProAtApx2* itself can initiate transcription of the *LUC* reporter gene. Furthermore, there exhibited brighter fluorescence when 1381ZLUC-ProAtApx2 and PMDC32-CtHsfA2b were co-transformed into protoplasts (lane 5) than 1381ZLUC-ProAtApx2 transformed alone (lane 4), which demonstrated that CtHsfA2b binding enhanced transcriptional activity of *ProAtApx2*. Fluorescence intensity measured by an ELIASA also showed a similar significant difference between lane 4 and 5.

## Discussion

In our study, *CtHsfA2b* was rapidly up-regulated within 1 h of 42 °C heat treatment and transcripts of *CtHsfA2b* attenuated quickly when plants were exposed to the normal temperature and remained at low levels during the recovery phase. These results confirmed that *CtHsfA2b* was heat inducible and could possibly trigger heat responses of downstream target genes quickly in the early stage of heat stress. Rice A2-type *HSF* genes (*OsHsfA2c*, *d* and *e*) also had quick heat response patterns similar to *CtHsfA2b*, while *OsHsfA2b* exhibited a different gradual increase of expression level under heat stress of 42 °C^16^. This difference implies that, due to the redundancy of the same type of HSFs, gene response patterns to HS may vary with the specific family member and plant species as well as the level of temperature stress^30^.

Feedback regulations are common in the complicated heat response pathways. After the heat shock peak, transcripts of *CtHsfA2b* attenuated quickly and remained at low level in the recovery stage in African bermudagrass. This may be due to the inactivation state of CtHsfA1. In previous studies of Arabidopsis and tomato, HsfA1 was activated by release from Hsp90/Hsp70 chaperone complexes as a result of triggering the transcription of HsfA2 in the initial stage of heat response that forms active homotrimers (HsfA1, HsfA2 and HsfB1)^31^,^32^. In the attenuation/recovery phase, transcription of HsfA2s is decreased by complex formation of HsfA1 with Hsp70 and the negative regulator heat shock factor binding protein1 (HSBP1) resulting in disintegration of active homotrimers to inactive monomers^10^,^33^. However, the transcript levels of the potential target genes of *CtHsfA2b* in transgenic *Arabidopsis* also attenuated rapidly within 2 h during heat treatment (Fig. 5), which indicated that the CtHsfA2b protein could become inactive as well with continuous heat stress. It can be deduced that the inactivation may due to interaction with Hsp17-CII, which was shown to act as a repressor for the transcription potential of HsfA2^34^, to form large cytoplasmic aggregates (heat stress granule, HSG). Furthermore, in the recovery stage, release of HsfA2 from HSG requires Hsp17-CI and probably the participation of Hsp70 and 101^10^,^34^. When heat stress re-occurs, the circulation is reinitiated, including changes between active HSFs triggering transcription and feedback regulation to inactive state^4^,^35^. *CtHsfA2b* had a typical characteristic of heat shock genes that are inducible by heat stress and become inactivated under normal temperatures to play a central role in the feedback regulation of HS.

HsfA2 is an important and irreplaceable regulator of a subset of heat-stress responsive proteins that maintain cellular stability and balance during heat stress and recovery stage^36^,^37^. The up-regulation of HsfA2 may markedly enhance transcriptional induction of a subset of HS genes, causing long-term heat protection effects^34^,^38^. In our study, the difference in phenotypes between transgenic lines and the WT plants was demonstrated after the relief of heat stress, even though the transcription of CtHsfA2b decreased to a low level in all plants in the recovery stage. In previous studies, overexpression of *OsHsfA2e* and *AtHsfA2* in *Arabidopsis* up-regulated 37 and 46 genes respectively^16^,^36^, and among these genes *Apx2* and *HSPs* also had elevated transcription levels. The induction of *CtHsfA2b* by heat stress could lead to subsequent induction of downstream genes with significant biological functions for heat adaptation, such as antioxidant enzyme AtApx2 for ROS scavenging and HSPs for protein protecting as discussed in detail below.

Heat stress induces oxidative damages due to excessive production of ROS^35^. Ascorbate peroxidase is one of the most important antioxidant enzymes in plants that detoxifies hydrogen peroxide using ascorbate as a reductant^39^, and *Apx2* has been considered the strongest HsfA2-dependent HS-responsive gene in *Arabidopsis*^38^. *Apx2* is one of the two cytosol isoforms of ascorbate peroxidase and another one is *Apx1*, which is considered a central factor of the reactive oxygen network, but *Apx1* is heat labile and its function might be compensated by *Apx2* under HS^38^. Transgenic *Arabidopsis* expressing *CtHsfA2b* exhibited higher expression levels of *AtApx2* and also had lower levels of hydrogen peroxide accumulation than the WT plants indicating improved antioxidant capacity during heat stress. In a previous study, *Apx2* mRNA levels also showed the strongest dependence on enhancement of all *APX* genes in HSF3-transgenic *Arabidopsis* plants^39^. We confirmed that *CtHsfA2b* was bound to the HSE on the *ProAtApx2* and could regulate its transcription level during heat response. Additionally, transcription activity may be enhanced by other HSEs in the *AtApx2* promoter or other transcription factors that interact with *CtHsfA2b*. *CtHsfA2b* may be an important HSF during elevated temperatures that induces antioxidant mechanisms to protect the plants.

Our results suggested that *CtHsfA2b* could be involved in enhancing heat tolerance of plants through regulating multiple HSPs [*AtHsp18.1-CI*, *AtHsp22.0-ER*, *AtHsp25.3-P and AtHsp26.5-P(r)*, *AtHsp70b and AtHsp101-3*], which have previously been shown to play important roles in heat tolerance. These HSPs were previously demonstrated as being targeted to the cytosol, chloroplasts and endoplasmic reticulum^2^,^38^, indicating the protection of most cellular compartments. Most of the up-regulated members by *CtHsfA2b* are small HSPs (sHSPs), whose monomers are only around 16–30 kDa but most need to form large oligomers (of 8 or more monomers) in the native state to function^40^, which may be the reason they were induced to very high transcription levels by heat stress. As an sHSP in chloroplast, Hsp25.3P (former name HSP21) was assumed to play important roles in protecting chloroplast stroma and facilitating protein refolding of photosystem II in tomato and *Arabidopsis*^41^,^42^. Hsp70 is a ubiquitous and important chaperone for normal plant growth and heat stress tolerance^40^, and Hsp101 was identified as a key component involved in resolubilizing protein aggregates for both basal and acquired thermotolerance in plants^43^,^44^. Additionally Hsp18.1 from pea (*Pisum sativum*) can stably bind heat-denatured proteins and maintain them in a folding-competent state for the further refolding by Hsp70/Hsp100 complexes^45^. Therefore, besides their respective important roles, the different classes of HSPs regulated by *CtHsfA2b* may cooperate in the cellular protection from heat stress.

In summary, the *CtHsfA2b* gene from heat-tolerant warm-season African bermudagrass was heat-inducible, and its ectopic expression in *Arabidopsis* up-regulated downstream genes involved in protein chaperone functions and antioxidant protection resulting in improved heat tolerance. The specificity and importance of *CtHsfA2b* lie in its rapid induction by heat stress, its strong accumulation and systemic transcriptional regulation on target genes. These results suggest that *CtHsfA2b* may be an important regulator of heat stress responses and the activation of heat tolerance mechanisms. *CtHsfA2b* could be potentially used as a candidate gene for genetic transformation or molecular breeding to improve heat tolerance in cool-season grasses and develop improved cultivars.

## Additional Information

**How to cite this article**: Wang, X. *et al*. Transcriptional regulation of heat shock proteins and ascorbate peroxidase by CtHsfA2b from African bermudagrass conferring heat tolerance in *Arabidopsis*. *Sci. Rep.* **6**, 28021; doi: 10.1038/srep28021 (2016).

## Supplementary Material

Supplementary Information
