# Supplementary material for: Transcriptional regulation of heat shock proteins and ascorbate peroxidase by CtHsfA2b from African bermudagrass conferring heat tolerance in Arabidopsis
Source: Sci Rep. 2016 Jun 20;6:28021. doi: 10.1038/srep28021 (PMC4913247; doi:10.1038/srep28021)
Supplement: Supplementary Information [file srep28021-s1.pdf]

Supplementary Information

Transcriptional regulation of heat shock proteins and ascorbate peroxidase by CtHsfA2b from African bermudagrass conferring heat tolerance in *Arabidopsis*

Xiuyun Wang [1, 2, †], Wanlu Huang [1, †], Zhimin Yang[1], Jun Liu [1, *] & Bingru Huang [2, *]

[1] College of Agro-grassland Science, Nanjing Agricultural University, Nanjing, 210095, China. [2] Department of Plant Biology and Pathology, Rutgers, the State University of New Jersey, New Brunswick, NJ, 08901, USA.

[†]These authors contributed equally to this work. [*]Correspondence and requests for materials should be addressed to B.H. (email: huang@aesop.rutgers.edu) or J.L. (email: liujun825@njau.edu.cn)

Supplementary Table S1. Information of primers involved in this study.

Primer Name	Primer Sequence (5’-3’)
CtHsfA2b-F	TGAGATGAGAAAGGAGCTCCAAGAGGCC
CtHsfA2b-R	CAACAAAACAAAATCAGTTGCTGCCTAAAGGT
CtHsfA2b-GSP	GATAGCCCATCTTCTCGGACAGCACAT
CtHsfA2b-NP	TGCTCTAGGCTGCCACTAGCACCCAT
CtHsfA2b-S	AATGGACCCAATGCTGAACCCG
CtHsfA2b-A	TGCTGCTTTGCTGTCACGGTAT
pEarleyGate103-R	TGATGCAACATACGGAAAACTTACC
LP	AAGGTTCCGAACCAAGAAAAC
RP	TCCTTCCACGTTACTTCAAGC
LBb1.3	ATTTTGCCGATTTCGGAAC
RT-*CtHsfA2b*-F	TATGGTTTCAGGAAGGTGGA
RT-*CtHsfA2b*-R	GGGCATTATGAGAAGGAGGT
AtACT2-F	TGCCAATCTACGAGGGTTTC
AtACT2-R	TTCTCGATGGAAGAGCTGGT
AtApx2-F	GGATGGGACTCAATGACAAAGATA
AtApx2-R	GTCGGTTGGTAGTTGAAGAAGTCC
AtHSP18.1-F	GGGAAGTTTATGAGAAGGTTTAGGTT
AtHSP18.1-R	CAAGCCAAGAAAAAAACACAAACT
AtHsp22.0-F	GCTTGAGAATGGTGTGCTCACTAT
AtHsp22.0-R	GGTAACTCTCTTCAGACTCAGAAAGTAATA
AtHsp25.3-F	AAACAGAGGAGGAAGTGGAGTGTCAGA
AtHsp25.3-R	AATCATCACTGTCTTCCTTCTTCTGCT
AtHsp26.5-F	CAAAGAGTTATGGTTACTACAACACGA
AtHsp26.5-R	ACGACACCGTATCTCTTCTACTCAA
AtHsp70-F	GAAGAGGTGGATTAGAGCGTGTTTAGT
AtHsp70-R	GACACAATACAAAGAAAAGACTCGCTA
AtHsp101-F	AAAATGCAACCTTTTGGAGCC
AtHsp101-R	TTCATAACCTCTGGACCTTTGAGAC
pHIS2 F	GCCTTCGTTTATCTTGCCTGCTC
pHIS2 R	CGATCGGTGCGGGCCTCTTC
ProAtApx2-LUC-F	TTCAATTGATCAATATGTCGAT
ProAtApx2-LUC-R	AGTTGTTACTTTAGTATACGGA

Supplementary Figure S1

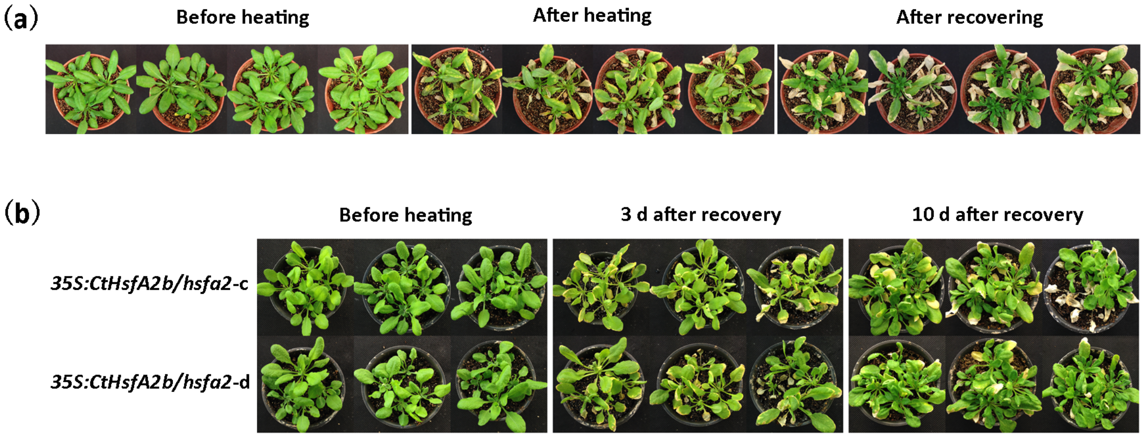

Supplementary Figure S1. Heat stress tolerance of transgenic plants. (a) Phenotype of the transgenic line *35S:CtHsfA2b*-c. The experiments were performed as described in Figure 4a. (b) Phenotype of the transgenic lines *35S:CtHsfA2b/hsfa2*-c and -d. The experiments were performed as described in Figure 6d.
